# Drivers of Consumer Liking for Beef, Pork, and Lamb: A Review

**DOI:** 10.3390/foods9040428

**Published:** 2020-04-03

**Authors:** Rhonda Miller

**Affiliations:** Department of Animal Science, Texas A&M University, College Station, TX 77843-2471, USA; rmiller@tamu.edu; Tel.: +1-979-845-3901

**Keywords:** consumer sensory, beef, pork, lamb, tenderness, juiciness, flavor

## Abstract

Tenderness, juiciness, and flavor have been associated with consumer acceptance of beef, lamb, and pork. Drivers of consumer liking are interrelated across these species, but there are differences in consumer preferences. Animal age, animal diet, and subsequent marbling impact consumer liking across species. For beef, consumer research prior to the 1990s showed that tenderness was the main driver of liking. Consumer tenderness and juiciness liking are highly correlated. More recent research has shown that as overall tenderness improved and tenderness variation decreased, flavor has become a more important driver of beef consumer liking. Flavor is affected by consumer preparation methods, familiarity with different flavor presentations, and animal production systems. Animal diet impacts consumer perception of beef tenderness and flavor, especially when comparing forage-fed versus grain-fed beef. Flavor preferences vary across countries more so than preferences for beef based on consumer tenderness preferences and are most likely influenced by the consumption of locally produced beef and the flavor-derived type of beef traditionally consumed. Drivers of pork consumer liking have been shown to be affected by pH, color, water holding capacity, animal diet, and the presence of boar taint compounds. While tenderness and juiciness continue to be drivers of consumer liking for pork, flavor, as impacted by animal diet and the presence of boar taint compounds, continues to be a driver for consumer liking. For lamb, the flavor, as affected by diet, and animal age continue to be the main drivers of consumer liking. Lamb consumers vary across countries based on the level of consumption and preferences for flavor based on cultural effects and production practices.

## 1. Introduction

Meat scientists have understood since the early 1900s that, in assessing and understanding meat eating quality, the end goal is to meet consumer demand and acceptance for meat products. In the early scientific literature, scientists eluded that systems such as the USDA Beef Quality grade system utilized factors that segmented beef carcasses into groups based on expected palatability. Meat scientists used trained descriptive sensory attributes for juiciness, tenderness, and flavor as indicators of consumer acceptability. However, while it was always implied that improvements in juiciness, tenderness, and flavor were associated with consumer acceptance, data were not presented [1,2,3,4]. The question was always present, “How are trained descriptive attributes related to consumer liking or acceptability?” While it is intuitive that trained human assessment of juiciness, tenderness, and flavor would most likely be related to consumer liking or disliking, it was not until Francis and others [5] that data were reported to understand consumer acceptance or liking of beef differing in USDA Beef Quality grades [6]. However, consumers were recruited from a farm show, and there was limited interpretation of the data. The main issue was that scientific methods in conducting consumer sensory evaluation that provided repeatable results were evolving. The American Society of Testing Materials published the first guidelines on sensory methods for foods and consumer products [7], and the American Meat Science Association (AMSA) published their first guidelines for cookery and sensory evaluation of meat [8], but these guidelines did not include an assessment of consumer sensory evaluation. It was not until 1995 that AMSA included consumer testing information in their sensory guidelines [9]. As methods evolved within the sensory community, scientifically accepted methodologies and guidelines for conducting and reporting consumer sensory data were developed [9,10,11]. Disciplines such as psychology, marketing and consumer insights, neural psychology, and sensory science used consumer sensory tools, but it was not until the 1980s that consumer sensory evaluation was used to understand meat product acceptance [12]. Much of the scientific community used trained descriptive panels to evaluate meat eating quality. Today, consumer sensory methodologies are used extensively by researchers to address acceptance of or preference for meat products. While consumer attitudes and familiarity with meat products play an important role in consumer liking and intent to purchase [13,14,15], these issues will not be directly addressed. Extensive research has also been conducted to examine pre- and post-harvest factors that impact consumer liking and meat eating quality. These papers will not be discussed, as it is not the intent of this paper. The effect of diet on meat flavor and sex on boar-taint in pork will be included in discussions, as these factors impact meat flavor, and pre- and post-harvest factors are important in providing road maps for methods to alter or improve tenderness, juiciness, and flavor of meat. The objective of this paper is to review research that evaluates consumer eating quality acceptance of whole muscle beef, lamb, and pork meat products, and to understand the current drivers of consumer liking. While the effect of meat color and visual assessment play a major role in consumer liking and purchase intent [13,16], this paper will only address the effect of eating quality on consumer liking.

## 2. Beef Consumers

The first extensive study that included consumers from multiple locations in the US was defined as the National Consumer Retail Beef study (NCRBS) [12,17]. In the NCRBS, the effect of marbling on consumer preferences for consumers in three cities was determined. The study included 540 households that contained two beef eating consumers. While they also conducted trained descriptive sensory evaluation for juiciness, tenderness, and flavor, they concluded that tenderness was the most important trained descriptive attribute driving consumer liking. While they showed that trained descriptive attributes showed similar trends to consumer data, they did not report relationships between trained and consumer sensory attributes. In the second phase of the NCRBS, they concluded that some consumers rated USDA Choice steaks higher for overall liking due to taste, and other consumers rated USDA Select steaks higher for overall liking due to leanness. This study provided evidence that consumer sensory research could provide valuable insight into beef acceptability and that consumer segments existed for whole muscle beef steaks.

The second national study was conducted in 1993 and 1994 and defined as the Beef Customer Satisfaction study. Beef top loin, top sirloin, and top round steaks were evaluated in an in-home placement study involving two beef consumers in each of 300 households in four cities [18,19,20,21]). Consumers were asked to cook steaks as they normally would prepare beef and to define the cooking method and degree of doneness. Consumers rated steaks for overall, tenderness, juiciness, and flavor liking. Descriptive sensory attributes and mechanical assessment of tenderness (Warner-Bratzler shear force; WBSF) were evaluated in companion steaks using a standardized cooking method. Relationships were examined between consumer and trained sensory measures and concluded that it was difficult to predict from descriptive sensory data how consumers would rate meat at home [22]. It should be noted that multivariate statistical tools were not used to evaluate relationships. As consumer data are more variable than trained descriptive data, multivariate statistical analysis tools for examining this relationship provide greater insight, and these tools have evolved since the 1990s. Additionally, tenderness and flavor liking were determined to be equal contributors to consumer liking for whole muscle beef steaks [18]. This was pivotal for the beef industry and meat science researchers as much of the focus for improving beef eating quality had been on improvement and assessment of beef tenderness. With the reporting of these results, the contribution of beef flavor to overall consumer liking was recognized.

In the 1990s and beyond, consumer research has become a highly utilized research tool to understand factors that affect meat eating quality and consumer liking. Research by meat scientists around the world has utilized consumer sensory evaluation to understand pre- and post-harvest factors that affect meat eating quality [23,24,25,26,27,28,29,30,31,32,33,34,35,36,37,38,39,40,41,42,43,44,45,46,47,48,49,50,51,52]. In Australia, consumer research has been the basis for the development of the Meat Standards Australia beef evaluation system [53,54,55,56,57]. This integrated system utilized pre- and post-harvest factors to predict the eating quality of individual beef cuts based on the cooking method. The MSA consumer evaluation methodology has been used to assess consumer liking across countries. Research using European [15,58,59,60,61,62,63,64,65,66,67,68,69], New Zealand [70], Asian [71,72,73,74,75,76], South African [77], and United States consumers [47,78,79,80] using the MSA consumer methods have been conducted. This research has established that consumers respond similarly to differences in tenderness, but flavor is more affected by cultural and environmentally learned behaviors in some countries. Consumer sensory studies that included factors and overall liking scores for beef across countries are presented in Table 1. While other studies have been reported, these selected studies provide evidence of factors affecting consumer overall liking.

**Figure 1:**
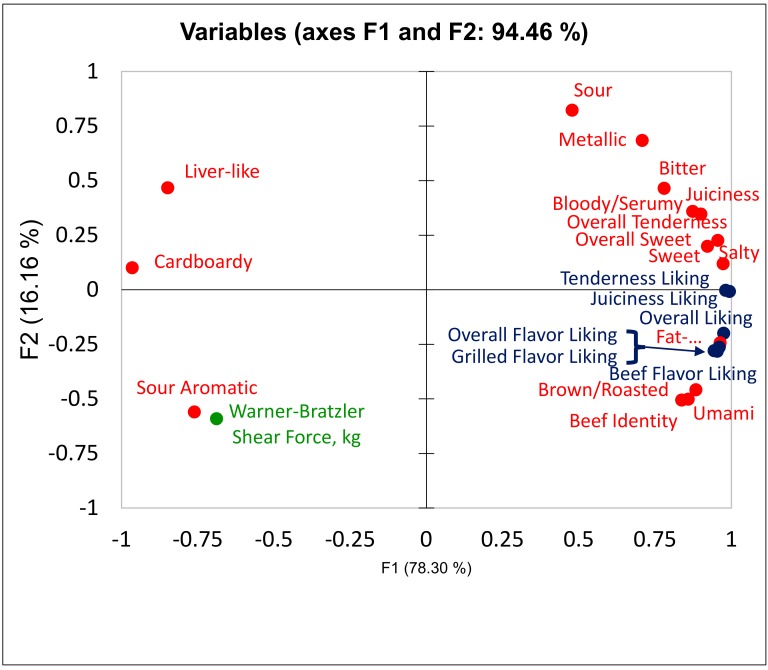
Principal component biplot of data adapted from Laird (2015) [90] and Luckemeyer (2015) [82] where descriptive flavor and texture attributes are in red, Warner-Bratzler shear force (WBSF) values are in green, and consumer sensory attributes are in blue.

A review of the MSA system and the research associated with the development and evaluation of the effectiveness of the system across different countries for beef and lamb has been presented [57]. Most of these studies indicated that tenderness liking is important to consumers, and consumers segmented whole muscle beef based on differences in tenderness. However, beef flavor liking is also a driver of consumer liking, and in some studies, flavor liking was a stronger driver of overall liking than tenderness liking [81,82,83,84]. This may be the result of improvements in overall beef tenderness and reductions in variability in beef tenderness. Within the US beef industry, beef in the retail meat case and in the foodservice industry has been monitored since 1989 to assess tenderness differences using WBSF, descriptive sensory attributes, and consumer sensory evaluation [23,85,86,87,88,89]. These surveys, known as the National Beef Tenderness Surveys, have shown that, for most whole muscle beef cuts, beef tenderness assessed using either WBSF, descriptive sensory panels, or consumer sensory panels has improved, and variation in beef tenderness has been reduced. The exception was for beef cuts from the round. With the advent of improved tenderness, it is not surprising that tenderness may not be as strongly related to overall consumer liking as in data from the 1980s, 1990s, and 2000s, where beef was tougher and more variable in tenderness. More recent consumer studies have shown that tenderness and juiciness are closely related, and as long as tenderness is within acceptable thresholds, flavor liking is the most prominent driver of consumer liking. Figure 1 presents a principal component biplot [82]. In these data, beef eaters who consume beef 1–2 times per week, defined as light beef-eaters, evaluated beef from 20 different treatments, where treatments included beef from different cuts cooked to two different degrees of doneness using different cooking methods to create differences in Maillard reaction products and lipid heat denaturation. Additionally, beef cuts were selected from USDA Select and Top Choice beef carcasses. Trained descriptive flavor and texture attributes were evaluated with an expert beef panel and consumers (*n* = 239) from three locations across the US in a central location consumer test. While beef differed in tenderness, least square means varied from 1.8 to 4.2 kg with a root mean squared error of 0.73. The biplot shows that tenderness and juiciness liking were closely related and not as closely related to overall liking as the three consumer flavor attributes were. WBSF was inversely related to consumer and trained descriptive attribute tenderness and juiciness measures, as would be expected, as higher WBSF values indicated increased toughness. Interestingly, trained descriptive flavor attributes of “fat-like” and “overall sweet” were closely related to overall liking or would be classified as positive flavor attributes, whereas trained descriptive flavor attributes of “cardboardy,” “liver-like,” and “sour aromatic” were negatively associated with overall consumer liking. This trend has been reported in multiple studies indicating that, as long as tenderness is acceptable, beef flavor attributes are currently the major driver for overall consumer liking. However, there is a caveat. Not all consumers have the same drivers for overall liking, and there are segments or clusters in most consumer data.

Beef is traditionally produced using either forages (defined as forage- or grass-fed), forages with grain supplementation, or high energy gran-based diets fed in the last 60 or more days prior to slaughter. For some consumers, the production system affects beef preferences based on personal beliefs or emotions in relationship to animal welfare, environmental issues, health, sustainability, and other factors [13,15,16]. The intent of this article is not to address psychological or marketing issues affecting consumer intent to purchase, but to concentrate on the meat sensory factors impacting consumer liking. The question is whether beef production systems, forage- or grass-fed versus grain-fed beef systems, impact the consumer perception of beef’s overall liking and the assessment of tenderness, juiciness, and flavor. Grass- or forage-based production systems are extensively used in Europe, Mexico, Central and South America, Africa, Australia, and Asia. While grain-based systems are the prevailing production systems for commodity beef in North America, grass-based beef production systems are evolving and becoming more prominent. Grass- or forage-based systems vary in the type of forage and quality of forage available for consumption, and extensive research has been conducted to understand beef production characteristics associated with quality of forage [93]. It has been well established that the quality of forage influences animal growth and the subsequent beef carcass characteristics. The question is whether beef derived from forage- or grain-based beef production systems impacts consumer liking.

It has been well established that beef produced on forage-based diets results in beef that is generally lower in total lipids [94,95]. Additionally, fatty acid composition can also be impacted; however, breed type and dietary forage can influence fatty acid composition [94,95]. Daley et al. (2010) and Van Elswyk and McNeill [94,95] reviewed research comparing the fatty acid composition of beef from forage- and grain-based production systems. In general, beef from forage-fed cattle is higher in saturated fatty acids, lower in monounsaturated fatty acids (mainly oleic acid), and higher in polyunsaturated fatty acids. The total amount of fat and fatty acid composition has been shown to affect the sensory characteristics of forage- and grain-fed beef [38,96,97,98,99,100,101,102,103,104,105,106,107,108,109,110,111,112]. In addition to changes in lipid content and fatty acid levels in forage- versus grain-fed beef, beef from forage-based systems has been shown to have higher levels of off-flavors compared to beef from grain-fed systems [94,96,97,113]. Elmore et al. [96] reported that grass-fed beef contained higher levels of diterpenoids—derivatives of chlorophyll called phyt-1-ene and phyt2-ene—that contributed to the flavor differences between grass- and grain-fed beef. The green odor found in grass-fed beef has also been associated with higher levels of hexanals derived from oleic and -linoleic acids [114]. In the United States, Canada, and Australia, beef consumers tend to like beef derived from grain-based production systems [38,78,91,101,103]. With increased exposure to beef from forage-based systems, larger consumer segments may develop preferences for beef from forage-based systems in the US. Japanese and South Korean consumers like highly marbled beef that is derived from grain-based beef production systems [72,74,75,108]. In other countries, varying results have been reported, and the drivers of liking differed across consumer segments. Beef from forage-based systems tends to be higher in lean-type flavors, such as beef identity, bloody/serumy, metallic, and liver-like, and lower in lipid-derived flavors, such as fat-like and cardboardy [38,114]. Oliver and others [115] showed that consumers in Germany, Spain, and the United Kingdom had different overall liking ratings when evaluating beef from forage-based systems in Uruguay versus locally grown beef from their respective countries. They identified three main clusters of consumers: consumers that preferred locally grown beef, consumers that preferred beef from Uruguay, and consumers that did not differentiate. In Chili, Morales et al. [59] reported that, in blind consumer tests, consumers in Chili liked beef from feedlot finished steers that had higher levels of marbling compared to both beef from pasture-fed low and high marbled beef and grain-fed low marbled beef. Garcia-Torres et al. [77] used beef from three production systems (organic beef fed on grass, organic beef fed on concentrate, and conventional concentrate feeding) in Spain. Spanish consumers segmented into two clusters. Cluster 1 mainly consisted of young and mature women from low to middle income levels with educational levels from secondary school to university studies. Consumers in Cluster 2 were mainly mature men with middle to high incomes with university education. Interestingly, consumers from Clusters 1 and 2, in an overall assessment, rated cooked meat lower for organic beef fed on grass compared to beef from either organic or conventional concentrate-fed treatments. Results from most of these studies are presented in Table 1**.** In summary, diets prior to slaughter for beef, either forage-, forage- and grain-, or grain-based diets, impact the sensory characteristics and consumer liking of beef; however, consumer segments for drivers of liking differ, and preferences are apparent across countries.

Extensive research has been conducted to examine other pre- and post-harvest factors that impact consumer liking or beef sensory properties. These papers will not be discussed, as extensive reviews have been published, though this research is important, as it provides factors that impact beef tenderness, juiciness, and flavor. It should be noted that, based on the data presented, factors affecting the eating quality of beef most likely will impact overall consumer liking.

While much of the meat science consumer research examines consumers who consume beef, pork, lamb, chicken, or other protein sources, the objectives of the research is usually to examine consumers of specific species. It should always be recognized that consumers who are beef consumers most likely are consumers of other protein sources. Therefore, the previous discussion of drivers of liking for beef most likely extend to pork and lamb consumers. However, pork and lamb consumers may have additional drivers of liking, and other factors may influence their overall liking. The following discussion will incorporate these additional drivers of consumer liking in addition to the aforementioned tenderness, juiciness, and flavor aspects.

## 3. Pork Consumers

Quality drivers of pork are generally recognized to be pH, water holding capacity, color, and marbling. Tenderness is considered a quality component for pork, but it has not always been evaluated. As pork is more variable in pH, water holding capacity, and color, how these factors influence consumer liking has been extensively examined across multiple countries and regions. In summarizing this research, pork that is high in pH is generally darker in color, has higher water holding capacity, is more intense in some flavor attributes, is juicier, and tends to be more tender than pork with a normal pH. Pork with a lower than normal pH has been shown to be lighter in color, have lower water holding capacity, be less intense in flavor, be drier, and be tougher than normal pH pork. Additionally, pork is traditionally cooked to higher degrees of doneness, and as low pH pork has lower water holding capacity, pork cooked to higher degrees of doneness tend to be tougher and drier. Extensive research globally has been conducted to examine these relationships.

In the United States, the last large comprehensive consumer study was to examine the effects of the aforementioned quality attributes on consumer perception [116]. Pork loins (*n* = 679) were selected to differ in pH, intramuscular fat, Minolta L* color values, and WBSF. Prior to serving to consumers, within the aforementioned parameters, pork was cooked to four endpoint cooking temperatures. The intent was to examine the main and interactive effects of the treatments. Consumers (*n* = 2280) were selected in three cities and were regular pork consumers. It should be noted that pork consumers are usually beef consumers as well. Data were analyzed using ordered logistical regression, and predicted mean consumer pork loin liking responses were presented. Consumer liking increased slightly as intramuscular fat levels increased, but increased levels of pH and decreased levels of WBSF influenced consumer liking ratings to a greater extent. Additionally, they found that, as internal cooking temperature increased, overall, tenderness and juiciness liking decreased [116]. They concluded that pork that had lower WBSF values, a higher pH, and intramuscular fat and that was cooked to lower internal temperature endpoints were liked to a greater extent by consumers.

Other consumer studies have examined factors driving consumer liking for pork. While the magnitude of the results may not be similar to those discussed above [116], consumer research has generally shown that pH, water holding capacity, tenderness as defined by either mechanical methods or trained sensory panel methods, intramuscular fat, and endpoint cooking temperature affect consumer liking [117,118,119,120,121,122,123,124,125]. However, pork production systems vary across some countries, and pork diets do differ in some areas of the world. In the United States, commodity pork is derived from animals fed soy bean- and corn-based diets. Additionally, live weight to slaughter varies by production system, and some counties castrate their males, while other countries traditionally slaughter intact males. These effects from production system/nutrition, live weight endpoint, and sex, mainly castrated versus non-castrated males, have been shown to affect pork sensory characteristics and consumer perception.

As pigs are monogastrics, flavor components from their diet can be absorbed and stored in the water portion or cytoplasm of adipose cells. Some of these compounds may be volatile aromatics and affect the flavor of the subsequent meat. The questions are whether consumers can detect these flavor aromatic compounds and, though they may not be able to describe the flavor, whether this change in flavor affects their overall liking. Research that examined changes in the energy level, grain source, supplementation sources, fat sources, and minor dietary constituents for pork has been presented [126]. While some dietary components influenced the pork’s flavor and lipid oxidation level, within the realm of standard high concentrate diets, little effect was found by adjusting small components of the diet. The adage that pork tastes like what the pig eats has some credence. Pigs fed specialty diets result in pork that generally has flavor attributes associated with that diet. The classic example is the production of Iberian pork meat in Spain, where pigs are fed acorns as a component of their diet. It has been well documented that fatty acid levels are affected by the diet and feeding system. For example, flavor and texture differences were apparent in meat from Iberian pigs fed on acorn and pasture versus confinement feeding [127]. Extensive research has also been conducted to examine breed type in pigs and the subsequent effect on consumer sensory perception. In general, breed or genetic types that produce pork that is higher in marbling and/or pH, such as Berkshire pigs for example, result in improved consumer perception.

Pork meat containing boar taint and the subsequent effect on consumer acceptability has been extensively examined [128]. Two main compounds have been associated with boar taint: androsterone (specifically androst-16-en-3-one) [129] and skatole (3-methylindole) [130,131]. Androsterone is a steroid hormone synthesized in the Leydig cells of the testis, and levels are affected by animal age, weight, sexual maturity, feeding and rearing conditions, herd factors, and genetics [132,133,134,135,136,137,138,139,140,141]. Skatole has a fecal odor and is the product of the anaerobic degradation of tryptophan in the gut. Its levels depend on the diet, rearing conditions, and handling of the pigs as well as sex, age, and genetics [134,142,143,144,145,146,147]. These compounds are mainly, but not solely, found in intact male pigs. With increased concerns over animal welfare, the practice of castration has been discontinued in some countries. It is generally accepted that as skatole and androsterone increase, consumers can detect off-flavors in pork, and consumer liking subsequently decreases [128]. The cooking procedure, the type of meat, and the age of the animal (intact males that have not shown signs of the onset of puberty) can affect consumer detection and levels of boar taint. Interestingly, consumers can vary in their detection of boar taint. The cooking method’s effect on masking boar taint in pork has been reported [148]. The most common thresholds for bore taint in pork are 0.5 and 1.0 μg/g for androstenone in fat tissue [134,149,150] and 0.10 and 0.20 μg/g for skatole in fat tissue [134,149,151,152,153]. However, approximately 40%–50% of consumers are anosmics or cannot detect androstenone [149,154,155,156]. Skatole on the other hand is perceived by 99% of consumers [157]. Research showed that consumer acceptability for pork with boar taint containing different levels of androstenone resulted in three consumer segments: pork lovers, boar meat lovers, and reject boar taint meat lovers [158]. Some studies have also shown that women are more sensitive than men to androstenone [154,155,159,160,161]. Immunocastration of pig has been proposed as a method to decrease the incidence of boar taint in pork with the subsequent strategy that immuocastration would decrease androstenone in intact males and increase consumer acceptance [162].

## 4. Lamb Consumers

Lamb meat across the globe has more variation in diet/nutrition and production parameters than pork and beef, resulting in more variability in lamb eating quality. Lamb is generally produced on either native forage, native forage with supplementation, or concentrate diets. However, more lamb is finished on forage-based diets than on concentrate diets globally. Feeding lambs concentrate diets is a common practice in the United States, but it is not a common practice in major lamb production counties of Australia, New Zealand, some European countries, and South America. Lamb flavor differs from beef and pork flavor and has mainly been reported based on differences in fatty acid content. When comparing lamb from animals fed on pastures versus lambs fed on high concentrate diets, differences in the fatty acid level and the subsequent differences in flavor have been reported [163,164,165,166,167]. In general, lambs fed on forage or pasture have more intense lamb, rancid, and liver flavors, as well as more intense levels of off-odors and off-flavors than lambs fed on concentrate diets. The type of forage also impacts lamb flavor [164,165,166]. The major fatty acid change in forage versus concentrate feeding is that forage-fed lambs have been shown to have higher levels of linolenic acid [168,169]. Linolenic acid is susceptible to lipid oxidation and off-flavors associated with lipid oxidation [170,171]. Caneque et al. [168] showed that pasture-fed lamb has been shown to have lower levels of linoleic acid [168]; linoleic acid is a component of lamb flavor identity [172,173]. While skatole has been associated with boar taint, meat from pasture-fed castrate and non-castrate lambs had higher skatole levels and may have a similar development of undesirable flavors in lamb as reported for pork [171]. It has been reported that increased skatole levels in lamb resulted in more intense sheep meat odor [163]. As in beef and pork, feeding diets with higher levels of grain or high concentrate diets result in lamb carcasses with higher fat levels and meat with higher levels of marbling [162,166]. Other research has shown that tenderness in lamb was related to marbling levels [174,175].

The question is whether differences in lamb eating quality induced by the feeding system affect consumer acceptability. Acceptance of Spanish, German, and French consumers for Uruguayan lamb fed a concentrate diet, a pasture-only diet, or two diets that consisted of a combination of pasture and concentrate diet was examined [167]. Consumers across countries preferred lamb fed the concentrate diet for overall, tenderness, and flavor acceptability. Similarly, Spanish consumers preferred the lamb fed concentrate diets [176]; whereas German consumers, even though they ate lamb less than once per month, preferred lamb from animals that had been fed a high concentrate diet [177]. Consumers in the UK preferred lamb that was from younger animals, and they indicated that they disliked intense mutton odor and flavor [178]. Mutton flavor has been associated with lamb from older animals and from pasture-fed animals. However, consumer clusters within country have been reported, and it was found that some consumers did prefer pasture-fed lamb, especially if the lambs had some supplementation of grain [167].

Lamb from Australia is predominantly derived from animals that are grass-fed and may have some grain supplementation [179,180,181]. Australian consumers did not distinguish lamb from animals fed pasture- or grain-based diets [182]. However, predominantly pasture-fed lamb was found to have a “pastoral” flavor [183]. “Mutton” flavor is traditionally associated with meat from older animals and has been identified in Australian lamb. Australian lambs are commonly harvested from 4 to 24 months of age, and older animals, defined as mutton, may also be harvested. Meat from older sheep or mutton has been shown to have detectable and higher levels of mutton flavor [184], and in general meat from older animals were less acceptable to consumers for tenderness, juiciness, flavor, and overall liking than meat from younger lambs [185]. Flavors defined as “pastoral” and “mutton” have been associated with increased levels of branched chain fatty acids. It has been reported that branched chained fatty acids most likely do not affect lamb flavor until lambs are over one year of age [186]. Levels of branched chain fatty acids were lower in animals less than one year of age, and the relationship between the level of branched chain fatty acids and lamb flavor was not found in younger animals. The relationship between the level of branched chain fatty acids and the intensity of pastoral and mutton flavors in lamb and subsequent consumer acceptance was examined [187]. They found that branched chain fatty acids of 4-methyloctanoic and 4-ethyloctanoic were related to consumer odor and overall liking attributes, but not to consumer flavor liking [187]. While other consumer studies have been conducted with Australian consumers and have reported a slight preference for grain-fed lamb [182,188], other drivers may influence consumer acceptance, such as frequency of consumption and environmental and health concerns [189].

Australian, Chinese, and United States consumers rated Australian lamb [57,189]. In general, consumers rated lamb loin and topside cuts similarly across countries, except for Chinese consumers, who rated topside cuts slightly lower in tenderness liking than Australian and United States consumers. Twelve different types of lamb produced in Greece, Italy, Spain, France, Iceland, and the United Kingdom were evaluated in a Home Use Test by consumers in these same countries [170]. Lamb was from animals that were either intact males, females or castrated males from 10 breeds and crossbreeds, fed pre-slaughter diets composed of either milk, concentrates, and various forages as main ingredients, of slaughter ages from 1 and 12 months, and with carcass weights from 5.5 to 30.4 kg. Family groups were identified that differed in their lamb preferences [190]. Those families with Mediterranean origin preferred lamb fed either milk or mainly concentrate diets, and families of mainly a northern origin preferred lamb from grass or with grass included in the diet. The remainder of consumers had a wider taste preference. Sanudo et al. [191] concluded that there are two categories of European lamb consumers of lamb: those who prefer a “milk or concentrate taste” and those who prefer a “grass taste.” While other consumer research has been conducted for lamb consumer liking, these studies provide some assessment of variation in taste preference for consumers in different markets.

In general, tenderness and juiciness are important to lamb consumers, as similarly identified for pork and beef consumers; however, flavor differences in lamb can impact flavor and overall acceptability. Consumer preferences for flavor are most likely affected by past experience in lamb consumption and cooking methods.

## 5. Consumer Segments

Consumer segments are extensively discussed in marketing and consumer insight research. It is well known that consumers today differ from consumers in the 1980s. The consumer segment defined as millennials, individuals born between 1977 and 1995, represent about 25% of the buying power in the US economy [192]. Millennials have been described as special, sheltered, confident, team-oriented, achieving, pressured, and conventional, and they are more numerous, more affluent, better educated, and more ethnically diverse than the previous major consumer segment, the baby boomers [193]. The US census identified millennials as the most diverse generation in American history, with 44.2% of millennials defined from a minority group [194]. Since millennials have had constant internet and social media access, they are more likely to be open to change and are more self-expressive than older generations [195]. Millennials use technology much more than previous generations did. Based on the changes in the environment for consumer segments, the question that Laird [83] addressed was as follows: Do millennials have the same drivers of overall liking as the combination of generation X and baby boomer consumer segments? It should be noted that generation X and baby boomer segments were combined. She recruited consumers in four cities that were either light (eat beef 2 or 3 times per month) or heavy (eat beef 3 or more times per week) beef eaters that were millennials or were older than millennials, which included generation X and baby boomer consumer segments. Consumers evaluated two different beef, chicken, and pork cuts in a Central Location Test (CLT) and in a Home Use Test (HUT). While millennials tended to cook beef steak, chicken breasts, and pork chops using pan-frying cooking methods more frequently than grilling, differences in consumer sensory attributes did not differ, except light beef eaters in both age groups had lower scores than heavy beef eaters (Table 2). Consumers from the four segments indicated that price was the first driver of meat purchase intent and that overall flavor liking was most closely related to overall liking for beef consumers, as similarly reported in Figure 1. Additionally, juiciness and tenderness liking were closely associated with each other and somewhat closely related to overall liking. Interestingly, consumers across the four segments rated the beef, chicken, and pork similarly, regardless of whether they were evaluated in the CLT or HUT portion of the study. These data demonstrated that, while there may be other drivers of purchase intent and of potential repeat purchases, demographics had little impact on consumer liking. It should be noted that consumer segments based on emotional or environmental concerns were not included. Therefore, questions on the drivers of liking for consumers who want, for example, locally grown, grass-fed beef without hormone applications was not addressed.

**Figure 2:**
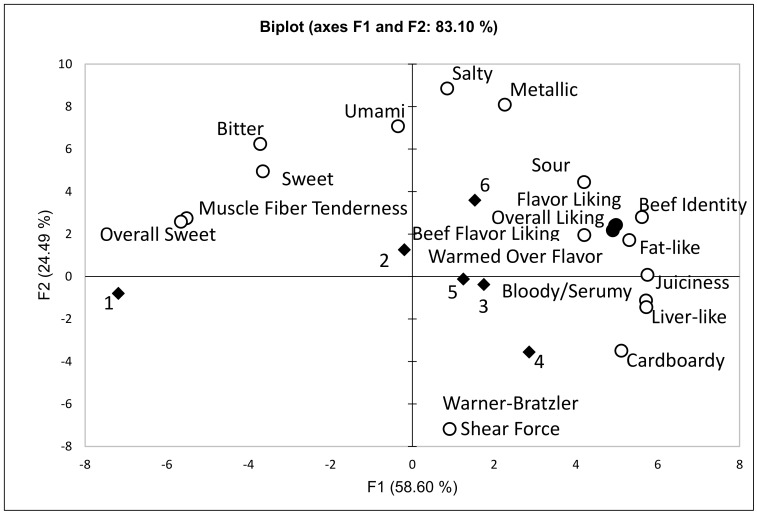
Principal component biplot of selected data from Laird (2015) [83] and Luckemeyer (2015) [82] using the six consumer liking clusters from Table 2. (◆), descriptive flavor and texture attributes and WBSF values (⚪), and consumer sensory attributes (⚫).

If consumer segments or demographics have changed, but are not related to consumer sensory perception, are there other consumer clusters or segments that may affect consumer perception? Consumer clustering techniques have evolved and are commonly used in marketing or consumer insight research. Statistical clustering techniques, such as k-means, agglomerative hierarchical clustering, Gaussian mixture models, and univariate clustering, have been developed to segment consumers into groups based on similarities defined by the model. These techniques have had minimal use in the meat science literature. In examining data where beef top loin, top sirloin, tenderloin, and beef bottom round roasts were evaluated in four cities with 3541 consumer observations, six consumer segments were identified using agglomerative hierarchical cluster analysis, where consumer overall liking, flavor liking, and beef flavor liking were used to develop clusters (Figure 2) [82,83]. The six clusters differed in overall, flavor, and beef flavor liking. The objective of this analysis was to see if consumers who rated beef samples differently across beef products had different drivers of liking.

Using principal component analysis, segmentations of clusters were reported (Figure 2). It is apparent that consumers in Cluster 1 were more closely related to muscle fiber tenderness, sweet, overall sweet, bitter, and umami and negatively related to WBSF. These consumers appear to be more driven by tenderness than by flavor attributes. Consumers in Clusters 3, 4, 5, and 6 rated beef with more emphasis on beef flavor attributes. The segmentation of consumers in Clusters 4 and 6 indicates that flavor attributes affected their liking ratings differently. Consumers in Cluster 6 rated beef samples with more intense amounts of beef identity, sour, fat-like, and warmed over flavor aromatics higher for overall liking and flavor liking. However, consumers in Cluster 4 rated beef that had more intense levels of bloody/serumy, cardboardy, and liver-like flavor attributes higher for overall and flavor liking, and a higher WBSF value did not affect their overall liking ratings. While these are general trends, this analysis begins to segment consumers, and it is apparent that consumers have different drivers for overall liking. Oliver et al. [115] reported three clusters when examining consumer liking for Uruguayan meat and locally sourced meat in Germany, Spain, and the United Kingdom. They were able to detect differences in consumer age and beef product usage across consumers segments within each country. They concluded that Uruguayan beef was acceptable in Germany and to a lesser extent in Britain and Spain. Borgogno et al. [13] used k-mean cluster analysis to segment consumers based on familiarity with different types of fresh meat. The reported two clusters for familiarity ratings where Cluster 1 was defined as the low familiarity segment and Cluster 2 was the high familiarity group. They examined the effect of label information, the liking of appearance, the liking of taste, and the expectations of fresh meat quality. Consumer clustered differed for some of these attributes. Garcia-Toirres et al. [77] also used cluster analysis to differentiate consumers based on their knowledge of organic production, the frequency of consumption of organic food, and sociodemographic variables. While there were only slight differences in consumer overall liking for three types of beef, there were differences in the relative importance of attributes associated with color, origin, production system, and price for beef. Morales et al. [59] calculated three clusters for consumers in Chile when evaluating beef from four production systems (pasture-low marbling; pasture-high marbling; feedlot-low marbling; feedlot-high marbling). Cluster 1 included consumers that typically eat lean beef and was 75% women. Cluster 2 was composed mainly of men over 40 and was characterized as high expectation consumers, and Cluster 3 was composed of high education level consumers. Bernues et al. [14] segmented Spanish consumers based on a survey into four clusters. Cluster 1 was defined as the traditional or conservative cluster and was characterized as containing consumers who liked to cook, did not like foreign meals, and considered planning meals important for family nutrition and health. Cluster 2 consumers did not like or spend time cooking and did not like changes in meals. The consumers in Cluster 3 had open attitudes toward innovation and were deemed adventurous. They liked to cook, tried foreign recipes, and frequently changed their meals. Cluster 4 contained the lowest number of consumers and was categorized as careless. These consumers had simple eating and cooking habits, did not like going to restaurants, ordered meat when eating out, and were mainly young men who lived in cities and had low incomes and low levels of education. While consumer liking attributes were not evaluated, these data showed that consumer segmentation affects consumer attributes about meat. Nie and Zepeda [196] segmented US consumers into four clusters: rational, adventurous, careless, and a fourth segment that had some characteristics of both conservative and uninvolved consumers. The segments exhibited significant differences in organic and local food consumption. It was apparent that consumers across the four clusters differed in lifestyle and food purchasing habits.

Font i Furnols et al. [197] used cluster analysis to segment Spanish, French, and British consumers into clusters based on country of origin, price, and feeding system for lamb meat. They showed that consumers in different clusters rated lamb differently for overall acceptability depending on the lamb production system of pasture, pasture + 0.6% live weight of concentrate, pasture + 1.2% live weight of concentrate, and concentrate and alfalfa. These studies show that consumer clustering can be a valuable tool in understanding drivers of overall liking. As consumers have their own unique attitudes, experiences, expectations, and lifestyles, additional information on drivers of overall liking can be obtained by the addition of these type of analyses.

## 6. Other Consumer Sensory Methods

As a community, meat scientists have traditionally used either Central Location or Home Use consumer testing methods to address consumer perception. Some researchers have branched out to work with either agricultural economists or consumer insight/marketing experts to understand purchase intent. While these methods have application and provide additional information on consumer perception, other methods used by psychologists, neural psychologists, and human behaviorists can assist meat scientists in further understanding consumer acceptance. It is generally accepted that 95% of consumer decisions are influenced by the subconscious. Regardless of the percentage, it is greatly recognized that the subconscious plays a major role in consumer perception. However, how do we as meat scientists measure subconscious effects and its impact on consumer acceptance? Research tools are available for assessing and further understanding consumer perception and what drives consumer liking from the human behavior aspect [198]. Human behavior tools assess different aspects of consumer cognitive processes and can be used to further understand consumer perception. They include eye tracking [199,200,201], electrophysical responses such as heart rate, blood pressure, and skin conductance, electrodermal activity or galvanic skin response, brain activity through electroencephalography [202,203,204,205], fMRI [206,207,208,209,210], and facial expression [211]. These methods measure emotional and physiological responses and provide additional information on how consumers perceive a product. Use of these tools may help meat scientists fully understand consumer segments and the environmental and social factors that drive consumer liking within segments.

One of the greatest challenges consumer sensory scientists face is the impact of environments on consumer preferences [11]. Consumers respond differently in Central Location tests where every aspect of the environment and product presentation is controlled versus consumer responses in Home Use tests where the consumer has the opportunity to handle the product and prepare it as it would normally be used in the home, and where the family environment may influence overall liking. It has been well documented that an environment impacts sensory responses. Augmented and virtual reality systems have been used to emulate an environment similar to the environment where consumers would consume the product [212,213]. They reported differences in consumer liking based on the environment. Incorporation and understanding of environmental factors may assist researchers in more accurately understanding consumer perception and provide additional research tools.

## 7. Conclusions

Consumers can detect differences in eating quality of meat products. Consumer preference and acceptance of meat products across whole muscle beef, pork, and lamb showed that consumers perceive differences in overall liking in meat products mainly using assessments of juiciness, tenderness, and flavor. In general, as long as tenderness and juiciness are at acceptable levels, flavor is the main driver of overall liking. It should always be understood that all three components influence overall liking. However, there are different drivers of overall liking across consumer segments. Consumers usually assess tenderness and juiciness together or similarly. For flavor, consumers differ in drivers of liking. Product usage or exposure, environmental factors, socioeconomic factors, and cooking methods, for example, can influence consumer liking and consumer flavor liking. Consumer research has been extensively used as a research tool since the 1990s, and Australia has based their MSA system on consumer research, indicating that consumer evaluation of meat products is a viable, repeatable research tool. New advances in statistical tools to understand consumer segments are being incorporated into meat science research, but further use of these tools may assist scientists in understanding drivers of consumer liking. Additionally, the use of human behavior research tools may assist meat scientists in further understanding consumer drivers of liking.

## Figures and Tables

**Table 1:** Selected studies examining consumer overall liking of beef.

Study and Treatments	Country for Consumer Selection	Consumer Liking Rating
Moreles et al. 2013 ^1^ [59]	Chili	
Pasture, low marbling		4.9 ^ab^
Pasture, high marbling		4.9 ^ab^
Feedlot, low marbling		4.6 ^b^
Feedlot, high marbling		5.2 ^a^
Garcia-Torres et al. 2016 ^2^ [77]	Spain	
Organic fed on grass		5.95 ^b^
Organic fed on concentrate		6.74 ^a^
Conventional production fed on concentrate		6.89 ^a^
Realini et al. 2013 ^3^ [90]		
Spain	Spain	
Grass-fed		5.66
Grass plus concentrate (0.6%) fed		5.83
Grass plus concentrate (1.2%) fed		5.59
Concentrate plus hay fed		5.43
France	France	
Grass-fed		5.53 ^ab^
Grass plus concentrate (0.6%) fed		5.63 ^a^
Grass plus concentrate (1.2%) fed		5.69 ^a^
Concentrate plus hay fed		5.11 ^b^
United Kingdom	United Kingdom	
Grass-fed		5.48 ^a^
Grass plus concentrate (0.6%) fed		5.67 ^a^
Grass plus concentrate (1.2%) fed		5.62 ^a^
Concentrate plus hay fed		4.98 ^b^
Killinger et al.^4^ [35]	United States	
High marbled beef		5.4 ^a^
Low-marbled beef		5.1 ^b^
Sepulveda et al. 2019 ^5^ [91]	United States	
Prime		67.8 ^a^
Top Choice		65.0 ^ab^
Low Choice		61.2 ^bc^
Select		59.6 ^c^
Bueso et al. 2018 ^4^ [92]	Hondurus	5.2 ^a^
	United States	4.0 ^b^
		US. Honduran
Grain fed, Select US beef	Values estimated	5.2 ^b^ 5.0 ^b^
Grain fed Top Choice US beef	From graph	5.8 ^a^ 5.1 ^b^
Honduran grass-fed, Bos indicus		5.0 ^b^ 3.5 ^d^
Honduran grain-fed		4.4 ^c^ 3.1 ^d^
Corbin et al. 2015 ^5^ [78]	United States	
Australian Wagyu (26.64%)		70.15 ^a^
American Wagyu (18.37%)		73.22 ^a^
Prime (14.67%)		71.58 ^a^
High Choice (8.99%)		61.24 ^b^
Top Choice, Holstein (8.54%)		62.67 ^b^
Low Choice (5.56%)		62.93 ^b^
Grass-finished (3.81%)		43.31 ^d^
Select, Holstein (3.45%)		50.40 ^c^
Select (3.31%)		50.95 ^c^
Standard (1.96%)		45.20 ^cd^
Van Wezemail et al. 2014 ^3^ [61]	Norway and Belgium	
WBSF 19–29.99 N		6.04
WBSF 30–40.99 N		6.08 ^a^
WBSF 41–51.99 N		5.16 ^ab^
WBSF 52–62.99 N		5.28 ^ab^
WBS 63–73.99 N		4.18 ^b^
McCarthy et al., 2017 ^5^ [68]	Republic of Ireland	
Irish beef		58.7
Australian beef		62.2
Chong et al. 2019 ^5^ [69]	Northern Ireland	55.6 ^a^
	Republic of Ireland	55.7 ^a^
	Great Britain	59.6 ^b^
Hwang et al. 2008 ^5^ [72]		
Grilling cooking method	Australian	63.5
Barbeque cooking method		66.2
Grilling cooking method	Korean	55.9
Barbeque cooking method		61.8
Bonny et al. 2017 ^5^ [66]	France	56.3
Only for consumers preferring medium degree of doneness	Ireland	54.0
	Northern Ireland	51.2
	Poland	55.6
Sitz et al. 2005 ^4^ [38]	Australian	4.34
	United States	5.37
	Canadian	5.49
	United States	5.79

^abc^ Within a study and column, means with the same letter are not significantly different (*p >* 0.05). Note that not all studies provided mean separations. ^1^ 1 = dislike extremely; 7 = like extremely. ^2^ 1 = dislike extremely; 10 = like extremely. ^3^ 1 = dislike extremely; 9 = like extremely. ^4^ 1 = dislike extremely; 8 = like extremely. ^5^ 1 = dislike extremely; 100 = like extremely. WBSF = Warner-Bratzler shear force.

**Table 2:** Least square means for consumer sensory attributes across consumer groups adapted from Laird (2015) [83].

Treatment	Overall Liking	Overall Flavor Liking	Beef/Pork/Chicken Liking	Grilled Flavor Liking	Juiciness Liking	Tender-ness Liking
*p-value*	*0.01*	*0.07*	*0.08*	*0.36*	*0.34*	*0.44*
Millennial, light beef eater	5.9^a^	5.8	6.0	5.4	6.3	6.2
Millennial, heavy beef eater	6.2^bc^	6.0	6.1	5.6	6.2	6.3
Non-millennial, light beef eater	5.9^ab^	5.9	5.9	5.5	6.2	6.3
Non-millennial, heavy beef eater	6.3^c^	6.1	6.3	5.6	6.5	6.5
Root Mean Square Error	2.21	2.23	2.22	2.34	2.28	2.27

^abc^ Mean values within a column followed by the same or no letter are not significantly different (*p* > 0.05).
